# Exploring policy driven systemic inequities leading to differential access to care among Indigenous populations with obstructive sleep apnea in Canada

**DOI:** 10.1186/s12939-015-0279-3

**Published:** 2015-12-18

**Authors:** Gregory P. Marchildon, Tarun R. Katapally, Caroline A. Beck, Sylvia Abonyi, JoAnn Episkenew, Punam Pahwa PhD, James A. Dosman

**Affiliations:** Institute of Health Policy, Management and Evaluation, University of Toronto, Toronto, Canada; Johnson Shoyama Graduate School of Public Policy, University of Regina, Regina, Canada; Indigenous Peoples’ Health Research Centre, University of Regina, Regina, Canada; Canadian Centre for Health and Safety in Agriculture, University of Saskatchewan, Saskatoon, Canada; Department of Community Health and Epidemiology, College of Medicine, University of Saskatchewan, Saskatoon, Canada

**Keywords:** Bifurcated health policy, Health inequities, Health care access, Settler nations, Indigenous Peoples, Status Indians, Health care systems, Canadian health care

## Abstract

**Background:**

In settler societies such as Australia, Canada, New Zealand and the United States, health inequities drive lower health status and poorer health outcomes in Indigenous populations. This research unravels the dense complexity of how historical policy decisions in Canada can influence inequities in health care access in the 21^st^ century through a case study on the diagnosis and treatment of obstructive sleep apnea (OSA). In Canada, historically rooted policy regimes determine current discrepancies in health care policy, and in turn, shape current health insurance coverage and physician decisions in terms of diagnosis and treatment of OSA, a clinical condition that is associated with considerable morbidity in Canada.

**Methods:**

This qualitative study was based in Saskatchewan, a Western Canadian province which has proportionately one of the largest provincial populations of an Indigenous subpopulation (status Indians) which is the focus of this study. The study began with determining approaches to OSA care provision based on Canadian Thoracic Society guidelines for referral, diagnosis and treatment of sleep disordered breathing. Thereafter, health policy determining health benefits coverage and program differences between status Indians and other Canadians were ascertained. Finally, respirologists who specialized in sleep medicine were interviewed. All interviews were audio-recorded and the transcripts were thematically analyzed using NVIVO.

**Results:**

In terms of access and provision of OSA care, different patient pathways emerged for status Indians in comparison with other Canadians. Using Saskatchewan as a case study, the preliminary evidence suggests that status Indians face significant barriers in accessing diagnostic and treatment services for OSA in a timely manner.

**Conclusions:**

In order to confirm initial findings, further investigations are required in other Canadian jurisdictions. Moreover, as other clinical conditions could share similar features of health care access and provision of health benefits coverage, this policy analysis could be replicated in other provincial and territorial health care systems across Canada, and other settler nations where there are differential health coverage arrangements for Indigenous peoples.

## Background

The Human Development Index consistently ranks Canada within the top five countries in the world, however, when the same methodology was modified and applied to Indigenous Peoples in Canada, they were placed at the 63^rd^ rank [1]. This gap resonates across the world, particularly in settler societies such as Australia, Canada, New Zealand and the United States, where Indigenous populations have lower health status and poorer health outcomes than majority populations [2]. While the causality for this phenomenon is complex, thus far the focus of research has been on determinants outside of the health care systems [3]. We have set out to investigate how historically rooted policy regimes determine current discrepancies in health care policy, and in turn how current health care policy drives health insurance coverage and physician decisions in terms of diagnosis and treatment.

Canada is divided into ten provinces and three territories, and health care in Canada is chiefly within the constitutional authority and responsibility of the provincial and territorial governments [4], with the federal government providing some standards for full coverage of medically necessary health services commonly known as Medicare [5]. Nevertheless, although provinces and territories are required to provide health services to their respective residents, significant health care access challenges exist for Indigenous residents of Canada.

During the period of European colonization of North America, the British Crown established a number of treaties with the some Indigenous groups of present day Canada [6]. Among those treaties, Treaty 6 includes a clause that states that all necessary medicine will be made available to those Indigenous groups and their descendants who signed the treaties in exchange for the surrender of vast land areas. With the Constitution Act declaring the formation of the Canadian Confederation under the British Monarchy [7], historically, some court decisions, and almost all Indigenous organizations and governments, have interpreted the clause in Treaty 6 as the federal government of Canada’s responsibility for providing health services to Indigenous Peoples [8]. However, since the provision of health services is a provincial and territorial responsibility in Canada, the health care coverage of many Indigenous citizens in Canada remains an ambiguous and inconclusive area of health care in Canada.

To further complicate this confusing status quo, the Canadian federal government classifies Indigenous Peoples based on their registration under the federal Indian Act [7]. Indigenous Peoples who are registered under this Act are classified as status Indians and those who are not registered are classified as non-status Indians [9]. Although the Indian Act itself is widely controversial and is considered to be a law that limits access to services and benefits for Indigenous peoples [9], the focus of this study is the exploration of status Indians’ access to diagnostic and treatment services.

Currently status Indians are entitled to receive full Medicare benefits as provincial residents; however, there exists a discrepancy when providing health care coverage for “extended health benefits”. Extended health benefits are all health services that are typically not covered by Medicare. In essence, unlike all other provincial residents whose coverage for extended health benefits is governed by provincial program benefits, status Indians are ineligible for provincial coverage. Instead they receive extended health benefits through the federally provided Non-Insured Health Benefits Program (NIHB). Apart from status Indians, Inuit populations are also eligible for NIHB. The Inuit traditionally lived above the tree line of current day Canada, and are part of a larger circumpolar Inuit population that includes Greenland, Alaska, and Russia [9, 10].

As status Indians and recognized Inuit receive extended benefits through NIHB, according to latest estimates, 925,000 status Indians and recognized Inuit (roughly 3 % of the Canadian population) are disqualified from receiving coverage through provincial and territorial extended health coverage laws and regulations [11]. The result is that in 13 different Canadian jurisdictions (i.e., 10 provinces and 3 territories) status Indians and recognized Inuit have different extended health coverage and conditions in comparison with other provincial and territorial residents. However, when it comes to Medicare, which includes medically necessary physician and hospital services, status Indians and recognized Inuit are treated no differently than all other provincial and territorial residents because the federal government insists that provincial governments provide such coverage in return for federal cash transfers for the financing of Medicare [5].

This bifurcation of extended health benefits coverage could influence physician decisions in terms of diagnostic and treatment approaches, and these physician decisions could lead to inequities in terms of disparities in access between status Indians and other Canadians in all 13 Canadian jurisdictions. To test this hypothesis, we identified a health condition that is associated with considerable morbidity in the Canadian population, and that requires extended health benefits coverage– obstructive sleep apnea (OSA).

OSA is the most common type of sleep apnea and manifests from blockage or collapse of the airways during sleep, thus causing episodes of apnea or hypopnea. Attempts at breathing during these episodes usually results in snoring, and overall, the condition is associated with poor sleep quality and excessive daytime sleepiness [12]. According to the Public Health Agency of Canada, an estimated 3 % (or approximately 850,000) of Canadian adults have been diagnosed with sleep apnea; however, more than 25 % of the Canadian adult population is estimated to be undiagnosed sufferers of the condition [13].

While OSA prevalence rates in the Indigenous populations are currently unavailable, it may be the case that the condition is more common among these groups due to experiences of poorer living conditions and lower socioeconomic status [14]. Indeed, previous research in the United States and Northern British Columbia identified a higher prevalence of low quality sleep among individuals of Indigenous descent [15, 16]. Moreover, OSA is associated with co-morbid conditions such as cardiovascular disease, diabetes, asthma, stroke, and metabolic syndrome and its components [17]. OSA is also associated with higher risk of motor vehicle accidents [18], low productivity due to excessive daytime sleepiness [19], and increased healthcare utilization patterns [20]. These associations of OSA with poor health outcomes places Indigenous individuals suffering from this condition at a higher risk of morbidity and mortality.

Studies of high-income countries indicate a consistently higher prevalence of OSA and sleep conditions in Indigenous and minority groups [21]. For example, the Maori people of New Zealand have been identified as having greater prevalence of insomnia, OSA symptoms and excessive sleepiness; greater severity of OSA symptoms; and poorer compliance with treatment compared to non-Maori people [21–25]. Similarly, in the United States, a study of older Medicare beneficiaries found greater sleepiness in non-Caucasian participants than their Caucasian counterparts [26].

In Canada, New Zealand and other countries, Indigenous populations’ over-represententation in lower socioeconomic groups [21, 23] could lead to potential access issues to specialized treatment and diagnostic services for sleep disorders and other respiratory conditions [22, 23, 27–29].

In this study we first explored health coverage discrepancies for OSA between status Indians and other residents in the Canadian prairie province of Saskatchewan (Inuit were excluded from this study because their traditional homelands are in the territories north of Saskatchewan). Thereafter, we delineated current Canadian and American diagnostic and treatment guidelines for OSA. Finally, we aimed to understand how health coverage and diagnostic and treatment guidelines for OSA influence physician decision making through interviews with respirologists based in Saskatchewan. Ultimately, this research is aimed to unravel the dense complexity of how historical policy decisions in a settler nation can influence inequities in health care access in the 21^st^ century.

## Methods

This qualitative study began with an extensive review of the secondary and the grey literature. First, Canadian Thoracic Society guidelines for referral, diagnosis and treatment of sleep disordered breathing were identified (Table 1). Thereafter, based on these guidelines, approaches to provision of care to patients with OSA were delineated. Finally, policy and program differences between the Saskatchewan government’s extended health benefits program and the federal government’s NIHB program in terms of providing care to patients with OSA were ascertained.

**Table 1:** Canadian thoracic society guidelines for the treatment and diagnosis of obstructive sleep apnea-hypopnea syndrome

Diagnostic Criteria	Referral	Testing Recommendation
Individual must fulfill criterion A or B, plus C (level of evidence D).	A. All patients who have suspected sleep-disordered breathing (SDB) should complete an assessment of daytime sleepiness such as the Epworth sleepiness scale (ESS) questionnaire to subjectively assess the degree of pre-treatment sleepiness.	Level I (complete laboratory polysomnography) remains the accepted standard for evaluation of SDB and is the test of choice (level of evidence C). However, a 2010 joint position paper from the Canadian Sleep Society and the Canadian Thoracic Society recommends the use of portable monitoring testing in three generalized settings for suspected sleep apnea in uncomplicated patients (i.e. without comorbidities) [37].
A. Excessive daytime sleepiness that is not better explained by other factors.		
	B. Patient referrals for assessment of SDB should be physician generated and should provide sufficient information to be able to determine the urgency of assessment.	
B. Two or more of the following that are not better explained by other factors:	C. Patients referred for medical specialist assessment and/or polysomnography should be triaged by the categories and criteria listed below.	
	Priority 1 (Urgent): Patients with suspected SDB and major daytime sleepiness (ESS of 15 or greater) and a safety critical occupation^a^ or patients with suspected SDB and a comorbid disease^b^ or overnight home oximetry that reveals greater than 30 oxygen desaturations (4 % or greater) per hour.	
		1. Areas with acceptable wait times for a sleep medicine consultation and Level 1 sleep study.
1. Choking or gasping during sleep;		
		2. Areas where the prevalence of Level 1 laboratory and sleep specialists are limited and the waiting times are excessive.
2. Recurrent awakenings from sleep;		
		3. Primarily rural areas, where sleep medicine specialists and Level 1 testing are not available, and where general practitioners (including nurse practitioners under the signing name of a physician) are the primary caregivers.
	Priority 2: Patients with suspected SDB and major daytime sleepiness (ESS of 15 or greater) but without a safety critical occupation.	
3. Unrefreshing sleep;	Priority 3: Patients with suspected SDB but without: major daytime sleepiness (i.e., ESS of 15 or greater), comorbid diseases, or a safety critical occupation.	
4. Daytime fatigue; and		
		Further recommendations and principles regarding the use of portable monitoring testing, including the accreditation of portable monitoring programs and technical and interpretation considerations are detailed in the joint position paper.
5. Impaired concentration.	Waiting times: Medical specialist assessment and/or polysomnography should be arranged and completed by the following times after referral (level of evidence D):	
C. Sleep monitoring demonstrates five or more obstructive apneas/hypopneas per hour during sleep.		
	• Priority 1 (urgent) cases – within two to four weeks;	
	• Priority 2 cases – within two months; and	
	• Priority 3 cases – within six months.	

^a^Safety critical occupations or at high risk for a motor vehicle collision

^b^Ischemic heart disease, cerebrovascular disease, congestive heart failure, refractory systemic hypertension, obstructive/restrictive lung disease, pulmonary hypertension or hypercapnic respiratory failure, or pregnancy

Sources: [30, 50]

This appraisal of current evidence was an integral part of the study methodology as it determined the path of inquiry to understand the intertwined policy and practice complexities of providing OSA care, including identifying and interviewing ten key informants. These key informants involved four Saskatchewan government extended benefit administrators, three federal government NIHB administrators and non-physician service providers, and three out of six respirologists in the province of Saskatchewan who specialize in sleep medicine care.

The inclusion criterion for the government administrators was their direct experience with the provincial or federal coverage policies and procedures related to OSA. The inclusion criteria for the respirologists included their specialization in sleep medicine and their current clinical practice in a publicly funded sleep clinic with a comprehensive sleep laboratory to test, diagnose and treat all Saskatchewan residents, including status Indians.

Ethics approval for this study was obtained from research ethics boards at the University of Regina and the University of Saskatchewan. Thereafter, informed consent for semi-structured interviews was obtained from all ten key informants. Interviews ranged from 25 to 75 minutes in length. With the exception of one interview conducted by telephone, all key informant interviews were conducted in-person. All interviews were audio-recorded and transcribed verbatim. Each participant received a transcribed version of their interview and was asked to review the content to ensure accuracy. At this point, each participant was given the option to withdraw the interview or any portion thereof based on the terms of the ethical consent agreement.

This study specifically focused on the perspectives of the three respirologists. The interview questions aimed to capture the complete cycle of provision of care to patients with OSA, and discern differential access to care between status Indians and other Canadians. The interview guide consisted of questions such as “On average, in a week, how often do you see patients in your office for the purpose of sleep-disordered breathing and/or OSA?”; “Are there common characteristics that you have observed in the OSA patient population that you treat, in terms of age, sex, weight, cultural background, or other characteristics?”; “In your opinion, what actions or changes in medical practice or health policy would make a significant impact on reducing the health impact of obstructive sleep apnea and sleep disordered breathing in Saskatchewan?”; “Are there specific actions or changes in medical practice or policy that would make a significant impact on OSA in Indigenous populations?” Thematic analysis was then conducted on the transcripts using NVIVO software and indicative coding techniques. The study results are a combination of evidence from secondary review of existing differences in extended health benefits coverage between status Indians and other Canadians, and emerging themes of physician decisions in terms of diagnosis and treatment of OSA.

## Results and Discussion

### Clinical diagnosis and treatment of obstructive sleep apnea

The Canadian Thoracic Society’s guidelines (Table 1) clearly indicate the referral, diagnosis and treatment protocols for OSA [30]. The diagnostic gold standard is a complete, level 1 polysomnography conducted in a specialized sleep laboratory. Patients are triaged to undergo polysomnography according to varying priority levels, thus resulting in waiting periods that range from two weeks to six months. The alternative to polysomnography is a level 3 home study, which involves the use of diagnostic equipment in the patient’s home and thus eliminates the waiting period. While lacking the specificity of polysomnography, a level 3 home study typically can diagnose the most common forms of OSA much more rapidly and at much lower cost.

Upon establishing an OSA diagnosis, the American College of Physicians recommends continuous positive airway pressure (CPAP) as the first course of treatment [31]. CPAP machine is a take-home system that requires the patient to wear a mask during sleep and delivers constant pressure flow to ensure the patient’s airway is maintained throughout the night. While CPAP is most commonly prescribed, adherence to CPAP treatment is a widely recognized issue and could result in poor outcomes due to lack of adherence leading to treatment inconsistency [32].

### OSA patient pathways

**Fig. 1:**
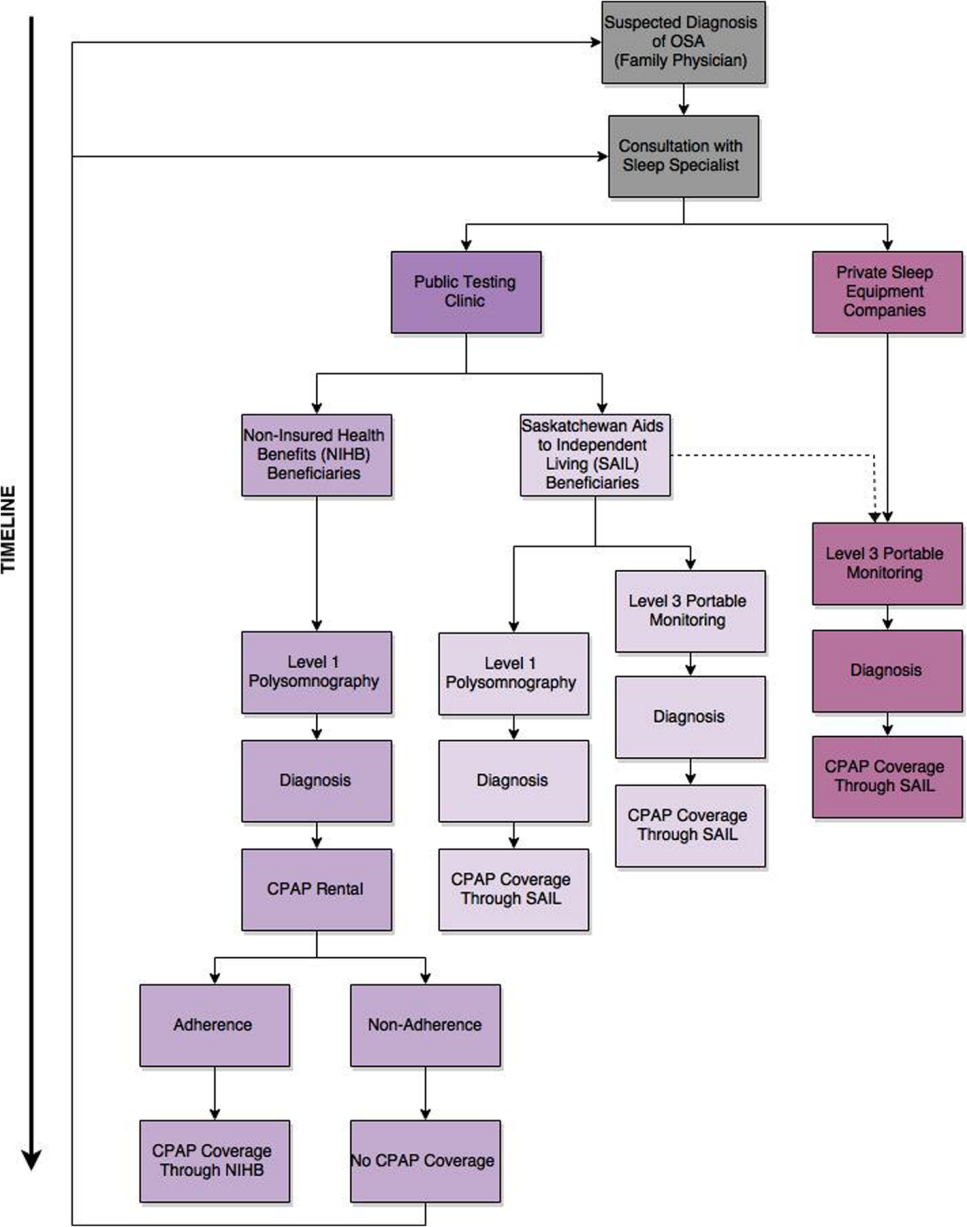
Patient pathways for obstructive sleep apnea diagnostic and treatment services in Saskatchewan. Note: Upon diagnosis, a patient with sleep apnea may decide to forego public coverage provided through NIHB or SAIL and instead purchase a CPAP machine directly form a private respiratory equipment provider

Figure 1 illustrates the potential OSA patient pathways in the health care system from the point of first patient-physician contact to diagnosis and treatment. Figure 1 also depicts the estimation of relative timeline difference in access to care between various pathways. Patients seeking care are first assessed by family physicians and if suspected to have OSA, are referred for consultation with respirologists specializing in sleep medicine. Health care until this point is provided by free provincial universally insured services to all residents, including status Indians.

However, after this stage, OSA care is provided through extended health benefits programs. Thus, provision of OSA care lies in a grey zone, with initial care (family physician and respirologist consultation) being provided through provincial Medicare plans and subsequent care (diagnosis and treatment) being provided through extended health benefits programs. In Saskatchewan, all residents except status Indians receive extended health care benefits through the provincial government’s Saskatchewan Aids to Independent Living (SAIL) program. As enumerated in the literature review, status Indians receive extended health benefits through the federal government’s NIHB program [33–36].

This separation of extended health benefits provision results in two distinct patient pathways. SAIL recipients (i.e., provincial residents other than status Indians) have shorter waiting periods as they have the choice of either level 1 polysomnography or level 3 portable monitoring for testing, and upon diagnosis, are eligible for CPAP coverage.

Based on existing policy documents, status Indians receiving extended health benefits through federally administered NIHB program incur considerably longer waiting periods, from initial testing and diagnosis to obtaining CPAP machines for treatment. This longer timeline for accessing care is the result of multilevel health inequities negatively influencing the health of status Indians. First, due to existing NIHB extended coverage policy, status Indians do not appear to have had the choice of level 3 monitoring for OSA diagnosis. Thus, they have to wait an average of 2 to 6 months to undergo level 1 polysomnography. Next, after diagnosis, status Indians are only eligible to rent CPAP machines initially through the NIHB program. NIHB requires patients to complete a 3-month CPAP rental period and provide proof of adherence to CPAP therapy before insuring CPAP purchase by status Indians. Finally, as there is evidence of adherence difficulties with CPAP treatment [34], accessing care for OSA through NIHB creates considerable barriers for status Indians. More specific differences in CPAP coverage conditions between SAIL and NIHB are further summarized in Table 2 [37–40].

**Table 2:** Coverage for CPAP machine including repairs and accessories for Saskatchewan residents

	Residents (except status Indians): SAIL	Status Indians residents: NIHB
CPAP machine	Free loan to patient on recommendation of respirologist (required level of testing not specified)	Purchase covered on recommendation of physician after Level 1 test and proof of adherence after 3-month CPAP rental period
CPAP repairs	Machine returned to SAIL depot in Saskatoon: free except for shipping costs	Coverage provided; however prior approval is required if warranty on device has expired.
Mask	No coverage provided^a^	Covered upon prior approval
Headgear	No coverage provided^a^	Covered upon prior approval
Tubing	No coverage provided^a^	Covered upon prior approval
Humidifier	No coverage provided^a^	Covered upon prior approval

^a^Additional coverage for these consumable supplies is available on a prior approval basis to those patients who qualify for the income-based Supplementary Health Coverage Program through Saskatchewan Health

Sources: [33, 38]

### Provider perspectives

Provider perspectives were probed in interviews with Saskatchewan-based respirologists practicing sleep medicine and whose practices involved both level 1 and level 3 diagnostic testing for Saskatchewan residents, including status Indians. At the time of the study, the three sleep medicine specialists located in Saskatoon were responsible for diagnosing and treating patients throughout Saskatchewan, a geographically expansive province with a significant percentage of its roughly 1 million residents living in rural and remote areas. While all respirologists provide services to status Indian patients, the proportion of status Indians accessing OSA care seemed lower in relation to the rest of the provincial population.

The three respirologists described that for every 15 to 20 patients who accessed the sleep lab, only 1 patient was status Indian, a proportion that is significantly below the 12.8 % of the provincial population (i.e., status Indians) who are deemed to be NIHB eligible clients by the federal government. In the words on one specialist, “there are less people referred to the sleep lab or to my office who are Status Registered Indian compared to non-Registered Indian. So my gut feeling, and I've not specifically done this research, but my gut feeling is they're not proportionally represented in either my office or the sleep lab compared to what the provincial population proportion should be”. One interesting result is that all three specialists initially felt that they did not treat patients any differently based on status and rejected any notion that they would treat patients differently based on ethnic origin or the terms of public or private coverage. At the same time, they all admitted that the administrative and coverage differences under NIHB could potentially produce at least some differences in their patterns of practice.

However, in thinking about the conundrum facing their patients, physicians did admit that approaches in dealing with status Indians was dictated in a more significant manner by the mode of coverage. One physician, when first asked whether his recommendation depended on the status of his patients, he stated that he did not treat his patients differently depending on the source of their coverage. However, upon reflection, he further reversed his earlier statement.

He went to say, “no actually, you know what? That’s not true. I’m going to withdraw that statement and the reason is that I know they [status Indians] won’t get funded for a CPAP machine if I send them for Level 3 testing, regardless who’s providing it, right? So I actually preferentially send people with a Treaty number (status Indian registration) for Level 1 polysomnography because I know that it’s going to be an administrative mess for them to try and get a CPAP machine if I don’t do that. It’s just a way of facilitating care for them.”

Another physician provided this answer to the same question, “My recommendation wouldn't vary, but as you know, because of Ottawa [the federal government], they want to have a Level 1 study done. So, does that affect how we care for people? Yeah, you have to do a level 1 study. Because of that policy, yes. And let's be honest. If they're from La Ronge (Northern Saskatchewan), it has nothing to do with if they're Status or not Status. It's a long long drive. If they're from Saskatoon, however, somebody who is Status versus someone who is non-Status, everything else being equal, they're going to have a longer wait because they have to wait for PSG.”

There was convergence among the three specialists on one key point. All agreed that, given existing times and the high public cost of Level 1 testing, Level 3 testing was a reasonable alternative, a school of thought that aligns with current diagnostic evidence [41]. All felt that, despite the evidence supporting this position for the majority of potential OSA patients, the federal government had not changed its administrative position. Moreover, while there was good reason to insist on adherence, two out of three respirologists felt that patient adherence to guidelines could be better supported through means other than using the threat of refusing treatment unless adherence was demonstrated – in other words, by treating status Indians the same as they would other Saskatchewan residents.

One physician summed up the issue surrounding adherence this way, “The First Nations group – at least the Treaty, you know, I guess I’m going to be specific here but the individuals with a Treaty number – are the only group where there’s some kind of deliverable attached to it, where they have to have used it a certain amount of time in order to get, or continue to use a machine. That doesn’t exist for other patients in Saskatchewan who use CPAP that’s provided by the payer – by Medicare – and it certainly wouldn’t exist for people who have gone out and bought their own CPAP machine, which is the other group. Not a very big group but it does exist.”

The findings from physician interviews validate the results from the review of diagnostic and treatment guidelines for OSA, and extended health benefits coverage. The current discrepancy in policy between the federal and Saskatchewan government’s extended health benefits for OSA care further perpetuates entrenched health inequities in terms of access to health care services. This discrepancy was encapsulated and a probable solution was suggested by one physician in this way: “You know, with respect to the First Nations community in particular, the goal of care of a person with a Treaty number – if you look at the big picture of, you know, “what is the federal government and the provincial government - what are they supposed to do here?” is that that person’s supposed to get treated the same way as any other resident of their community with respect to health care. So, in Saskatchewan, that would mean that they would have more timely access to a CPAP machine. Like I can write, I can get one for a patient, for example in the sleep lab, they get one same day of their assessment as long as they don’t have a Treaty number. If they have a Treaty number, I have to send them to a vendor and then they have to borrow a machine for a while and then they have to prove they’re using it and then… you know? There’s often big gaps for these people in terms of timely treatment. Sometimes much longer than you might expect – sometimes weeks and sometimes months. So a change where that, you know… it could be very simple. There’s a simple solution here – that SAIL (pardon me - Saskatchewan Aids to Independent Living) takes on the role of providing that to the First Nations community and then bills NIHB or invoices them or however you wanted to do that”.

In summary, the two major difficulties faced by the status Indian populations living on reserves in Saskatchewan [42] are geographic distances to the only two publicly funded sleep clinics (located in Saskatoon and Regina) and the mandatory level 1 polysomnography resulting in wait times of over a year due to the limited availability of sleep clinics in Saskatchewan [43]. The multi-level barriers to access to care could contribute to the underrepresentation of status Indians’ in sleep clinics, as described by the respirologists. This underrepresentation has wider implications in underestimating the prevalence of OSA among status Indians and as a consequence increases their risk of morbidity and mortality from comorbidities of OSA [12, 44–46]. With status Indians already suffering from higher prevalence of comorbidities of OSA [5, 47–49], an underestimation of OSA prevalence is indicative of how policy-driven health inequities can further widen existing health inequalities in disadvantaged populations.

### Limitations and strengths

While the interviews clearly reflected the view that status Indians were under-represented relative to the majority population in terms of the diagnosis and treatment of OSA, this qualitative research cannot establish that current policy is linked to the underestimation of OSA prevalence in status Indians in Saskatchewan. Moreover, this research is limited to only one jurisdiction in Canada and although clear patterns of disparities in coverage emerged, the findings can only be generalized to provinces with similar extended health benefits policy regimes. Nevertheless, this study is a novel investigation exploring policy driven practice that has implications not only for OSA patient care, but also for other clinical conditions that share similar features of lying at the boundary between universal Medicare coverage and the more fragmented systems of extended health benefit coverage across Canada.

## Conclusions

OSA is serious public health issue affecting all Canadians; however in Saskatchewan, considerably greater barriers faced by status Indians in accessing OSA care could ultimately influence underestimation of OSA prevalence in these populations. To understand if the implementation of federal government’s NIHB program in 13 sub-state jurisdictional settings in Canada in terms of providing OSA care coverage can differ depending on the configuration of provincial and territorial extended benefits programs, a more comprehensive study spanning all Canadian jurisdictions is necessary. However, before conceptualizing a complex cross-Canadian study, it is imperative to validate current findings with a longitudinal cohort study that compares not only access to OSA care, but also adherence to OSA treatment and clinical outcomes between status Indians and other residents in Saskatchewan.

## Abbreviations

CPAP: Continuous positive airway pressure
ESS: Epworth sleepiness scale
NIHB: Non-Insured Health Benefits
OSA: Obstructive sleep apnea
SAIL: Saskatchewan Aids to Independent Living
SDB: Sleep-disordered breathing
